# Gender differences in the use of modern nicotine products among adolescents in northern Germany: a cross-sectional school-based study

**DOI:** 10.1016/j.pmedr.2026.103676

**Published:** 2026-10-29

**Authors:** Mareike Lüthgen, Florian Graf, Niel Heitmann, Adrian Heinz, Daniel Drömann, Folke Brinkmann, Klaas F. Franzen, Loana Penner

**Affiliations:** a University of Lübeck, Medical Clinic III (Pulmonology), University Hospital Schleswig-Holstein, Campus Lübeck, Germany; b University of Lübeck, Department of Pediatrics, University Hospital Schleswig-Holstein, Campus Lübeck, Germany; c Airway Research Center North, Member of the German Center for Lung Research (DZL), 22927 Großhansdorf, Germany

**Keywords:** Adolescents, Nicotine products, e-cigarettes, Nicotine pouches, Gender differences, Poly-use, School-based survey, Prevention

## Abstract

**Objective:**

To examine gender differences in the use of cigarettes, e-cigarettes, and oral nicotine products among adolescents in Germany.

**Methods:**

This cross-sectional study analyzed survey data from 958 students aged 11–21 years at six secondary schools in northern Germany, collected February – May 2026 within the SchoolVapeScience project. Lifetime and regular use, and, among lifetime users, initiation, use patterns, consumption contexts, and motives were compared between male (*n* = 446) and female (*n* = 505) participants using chi-square tests, *t*-tests, and ordinal logistic regression.

**Results:**

Female participants reported higher lifetime (28.1% vs. 21.5%) and regular (17.0% vs. 10.3%) e-cigarette use, whereas regular use of oral nicotine products was more common among males (5.2% vs. 1.6%). Cigarette use did not differ by gender. Male users initiated e-cigarette use earlier (13.2 vs. 14.2 years) and more often reported poly-use (38.2% vs. 22.2%). Among cigarette users, weight control was a more common motive among females (21.1% vs. 1.5%), whereas male users of oral nicotine products more often reported school-related consumption contexts.

**Conclusions:**

Gender differences in adolescent nicotine use lie less in overall use than in product choice, use patterns, and motives. Prevention strategies should be gender-sensitive and address modern nicotine products.

## Highlights

• Gender differences in nicotine use reflect product choice, not prevalence.
• Female adolescents show higher e-cigarette use than male peers.
• Male adolescents use oral nicotine products more frequently.
• Males use more nicotine product types; females prefer single products.
• Weight control motivates nicotine use more often in female adolescents.

## 1 Introduction

The consumption of nicotine-containing products is currently undergoing substantial change. While the use of conventional tobacco cigarettes has declined in many Western countries, modern nicotine products such as e-cigarettes, heated tobacco products, and oral nicotine products including nicotine pouches and tobacco pouches (“snus”) are becoming increasingly popular, particularly among adolescents and young adults (Hanewinkel and Hansen, 2025; Hanewinkel and Hansen, 2023). These products are often marketed as modern, socially acceptable, or supposedly less harmful alternatives to smoking (Truth Initiative, 2026; Posch et al., 2026) and are closely linked to digital youth cultures, social media, and lifestyle-oriented consumption practices (Trümpelmann et al., 2026).

Historically, smoking was long considered a predominantly male-associated behavior. During the 20th century, however, smoking prevalence among men and women increasingly converged (Deutsches Krebsforschungszentrum and Deutsche Krebshilfe, 2025). Particularly following the targeted marketing of cigarettes toward women, smoking became partly associated with freedom, independence, and emancipation (Deutsche Hauptstelle für Suchtfragen e. V, 2002). Current epidemiological data indicate that gender-specific differences in nicotine consumption still exist but are changing over time. Whereas earlier research primarily focused on overall smoking prevalence, current differences appear to be more strongly related to product type, motives for use, and consumption patterns (Dalmau, 2021).

Recent research suggests that modern nicotine products are used by adolescents across genders, although differences in product preferences, frequency of use, and motivational factors have been observed. Studies report higher levels of risk-oriented behavior, greater poly-use, and more frequent use of oral nicotine products among male adolescents (Tattan-Birch et al., 2022; Sullman et al., 2024). Female adolescents more often report emotional or body-related motives for use, such as stress regulation or weight control (Hanewinkel and Hansen, 2023). However, it remains insufficiently understood to what extent traditional gender differences are changing or diminishing in the context of modern nicotine products and broader social changes.

While gender differences in conventional cigarette smoking have been extensively studied, less is known about whether these differences persist, diminish, or transform in the context of modern nicotine products among adolescents.

This question is particularly relevant given the increasing availability and attractiveness of new nicotine products. *E*-cigarettes, oral nicotine products, and related products differ not only in terms of health risks, but also with regard to design, marketing, social meaning, and contexts of use (Truth Initiative, 2026). At the same time, traditional gender roles are becoming less pronounced, potentially contributing to the emergence of new gender-specific consumption patterns that differ from those observed for conventional smoking behaviors.

Most evidence on gender differences in modern nicotine product use originates from the United States and the United Kingdom (Tattan-Birch et al., 2022; Cook et al., 2023; Harlow et al., 2025; Abid et al., 2025), where regulatory frameworks differ considerably from those in Germany. In Germany, the sale of snus is prohibited under the Tobacco Products Act, which implements the EU ban on oral tobacco. Tobacco-free nicotine pouches likewise cannot legally be sold, as nicotine is not authorized as a food ingredient under the EU Novel Food Regulation. In addition, the Youth Protection Act prohibits the sale of all nicotine-containing products to minors. Nevertheless, these products are used by German adolescents (Hanewinkel and Hansen, 2025), while data on gender-specific use patterns in this regulatory context remain scarce. The present study was conducted within an ongoing school-based research and prevention project in northern Germany.

The aim of the present study was therefore to examine gender-specific differences in the use of modern nicotine products among adolescents. The study focused on differences in prevalence, frequency of use, initiation patterns, motives for use, consumption contexts, as well as dual- and poly-use of different nicotine products. Given the low prevalence of current use, analyses beyond prevalence were based on lifetime users of the respective product.

## 2 Methods

### 2.1 Study design and population

This cross-sectional study was conducted within the School*Vape*Science project, a school-based educational and research initiative on adolescent nicotine use. In 2026, six secondary schools from urban, suburban, and rural regions in northern Germany participated in the study, including both academic-track and comprehensive schools to reflect socioeconomic heterogeneity.

Data collection took place prior to the delivery of standardized nicotine prevention sessions in order to minimize immediate intervention-related bias. The questionnaire was revised annually to reflect developments in the nicotine product market and included items on nicotine use behaviors, oral nicotine use, motives for use, risk perception, and social media exposure. The present analysis is based on the 2026 wave (February to May 2026). The survey was developed using LimeSurvey® software and pilot-tested within student groups involved in the project. No standardized instruments were used.

All students enrolled in grades 5–12 at participating schools were invited to participate voluntarily and anonymously. A total of 1007 students completed the survey in 2026. Of these, 49 participants were excluded because fewer than 50% of items were completed or response patterns were implausible, resulting in an analytical sample of 958 participants. Seven participants reporting diverse gender were included in descriptive analyses only, so gender-specific analyses were based on 951 participants. Participants did not receive any compensation.

### 2.2 Measures

Data were collected digitally via QR code-based survey access using tablets and smartphones. Students completed the questionnaire individually during supervised classroom sessions. The survey assessed sociodemographic characteristics, nicotine use behaviors, oral nicotine use, motives for use, perceived health risks, social and parental influences, media use, and self-reported health indicators.

Gender was assessed by self-report, with participants selecting one of three response options (male, female, or diverse). As the item captured self-identified gender rather than sex assigned at birth, the term “gender” is used throughout this manuscript.

Lifetime use was assessed for cigarettes, e-cigarettes, snus, and nicotine pouches (combined as oral nicotine products) using the question “Which of the following have you ever used?”. Additional product-specific items assessed age at initiation, consumption contexts (multiple response: alone, with friends, with family, at parties, at school, before exams or tests, and, for oral nicotine products, before or during sports), and motives for use; participants without lifetime use rated reasons for non-use. Motives and reasons for non-use were rated on a five-point scale from 1 (“does not apply at all”) to 5 (“fully applies”). Among lifetime users, frequency of use was assessed for each product (“How often do you use …?”) with five categories: “tried it once”, “occasionally (more than once a month, but less than once a week)”, “often (one to three times a week)”, “regularly (more than three times a week, but less than daily)”, and “daily”. This item referred to participants' usual pattern of use; no fixed recall period (e.g., past 30 days) was specified. Regular use was defined as any response other than “tried it once”, i.e., use more than once a month. No product images were shown.

### 2.3 Statistical analysis

Descriptive analyses were performed for all relevant variables. Categorical variables are presented as absolute frequencies and percentages. Missing values and structurally missing responses resulting from filter questions were excluded from the respective analyses. No a priori sample size calculation was performed; the study used a census-based approach enrolling all eligible students at participating schools.

For analyses of gender differences, only participants reporting male or female gender were included (*n* = 951). Responses of participants indicating diverse gender (*n* = 7) were reported descriptively only due to the small subgroup size.

Lifetime prevalence of nicotine product use, including conventional cigarettes, e-cigarettes, and oral nicotine products, was analyzed descriptively. Oral nicotine use included both tobacco and nicotine pouches. Participants reporting use of both product types were counted only once.

Analyses of first product used, age at first use, and nicotine use patterns were restricted to participants with lifetime use of at least one product. Analyses of consumption contexts and motives were restricted to lifetime users of the respective product.

Gender differences in prevalence, frequency of use, first product used, consumption contexts, and dual- and poly-use patterns were analyzed using chi-square tests. Effect sizes were assessed using Cramér's V. Expected cell frequencies were examined, and findings involving expected counts below five were interpreted with caution.

Frequency of use was treated as an ordinal variable. Ordinal logistic regression models were calculated to examine associations between gender and frequency of use while controlling for age. Results are reported as odds ratios (ORs) with 95% confidence intervals (CI).

Motivational items were assessed using five-point Likert scales ranging from 1 (“does not apply at all”) to 5 (“fully applies”). For analysis, responses were grouped into disagreement (1–2), neutral (3), and agreement (4–5). Gender differences were analyzed using chi-square tests.

Dual- and poly-use were defined based on the number of lifetime-used product categories. Single use referred to use of one product category, dual use to two product categories, and poly-use to three or more product categories.

A significance level of *p* < .05 was applied for all inferential analyses. Given the exploratory nature of the analyses, no formal adjustment for multiple testing was applied.

Statistical analyses were conducted using R (version 4.5.1).

### 2.4 Ethics statement

The study was conducted in accordance with the Declaration of Helsinki and approved by the Ethics Committee of the University of Lübeck (reference number: AZ 2024–267). Written informed consent was obtained from both participants and their legal guardians. All responses were anonymized.

## 3 Results

The total sample comprised *N* = 958 adolescents and young adults aged 11 to 21 years (M = 14.86, SD = 2.21). Participants were distributed across all age groups (≤ 13 years: 29.2%; 14–15 years: 28.3%; 16–17 years: 28.7%; ≥ 18 years: 13.8%). In total, 446 participants identified as male, 505 as female, and seven as diverse.

### 3.1 Lifetime prevalence and frequency of use

Lifetime prevalence rates of the investigated nicotine products are presented in Table 1 and Fig. 1. No gender difference was observed for lifetime cigarette use. Female participants reported significantly higher lifetime prevalence of e-cigarette use than male participants, whereas lifetime use of oral nicotine products did not differ by gender.

**Table 1:** Sample characteristics, lifetime and regular use, initiation patterns, and nicotine use patterns of cigarettes, e-cigarettes, and oral nicotine products by gender among secondary school students (grades 5–12) in northern Germany, February–May 2026.

	Male	Female	Statistics
	*n = 446*	*n = 505*	

*Sample characteristics*			
Age in years, mean (standard deviation)	14.8 (2.2)	14.9 (2.2)	–

*Lifetime use, n (%)*			
Cigarettes	78 (17.5)	91 (18.0)	χ^2^(1) = 0.02, *p* = .898, Cramér's *V* = 0.00
E-cigarettes	96 (21.5)	142 (28.1)	χ^2^(1) = 5.14, *p* = .023, Cramér's *V* = 0.07
Oral nicotine products	44 (9.9)	42 (8.3)	χ^2^(1) = 0.52, *p* = .473, Cramér's *V* = 0.00

*Regular use, n (%)*			
Cigarettes	38 (8.5)	43 (8.5)	χ^2^(1) = 0.00, *p* = 1.000, Cramér's *V* = 0.00
E-cigarettes	46 (10.3)	86 (17.0)	χ^2^(1) = 8.38, *p* = .004, Cramér's *V* = 0.09
Oral nicotine products	23 (5.2)	8 (1.6)	χ^2^(1) = 8.49, *p* = .004, Cramér's *V* = 0.10

*Participants with lifetime nicotine use (male: n = 102; female: n = 153)*ᵃ			
First nicotine product ever used, n (%)ᵇ			χ^2^(2) = 0.37, *p* = .832, Cramér's *V* = 0.00
Cigarettes	22 (22.7)	36 (24.8)	
E-cigarettes	72 (74.2)	107 (73.1)	
Oral nicotine products	3 (3.1)	3 (2.1)	

Age at first use in years, mean (standard deviation)			
Cigarettes	13.7 (2.5)	14.2 (2.1)	*p* = .184
E-cigarettes	13.2 (2.2)	14.2 (1.7)	*p* = .001
Oral nicotine products	15.1 (1.6)	15.7 (1.2)	*p* = .081
Nicotine use pattern, n (%)			χ^2^(2) = 11.15, *p* = .004, Cramér's *V* = 0.19
Single use	25 (24.5)	65 (42.5)	
Dual use	38 (37.3)	54 (35.3)	
Poly-use	39 (38.2)	34 (22.2)	

Oral nicotine products included snus and nicotine pouches; participants reporting use of both were counted once.

Regular use was defined as use more than once a month.

Single, dual, and poly-use refer to lifetime use of one, two, or all three product categories (cigarettes, e-cigarettes, oral nicotine products).

*P*-values for categorical variables were obtained from chi-square tests; *p*-values for age at first use were obtained from Welch's *t*-tests. Effect sizes are presented as Cramér's *V*.

ᵃ Percentages refer to male and female participants with lifetime nicotine use, respectively.

ᵇ Analyzed among participants reporting cigarettes, e-cigarettes, or oral nicotine products as their first product (male: *n* = 97; female: *n* = 146).

**Fig. 1:**
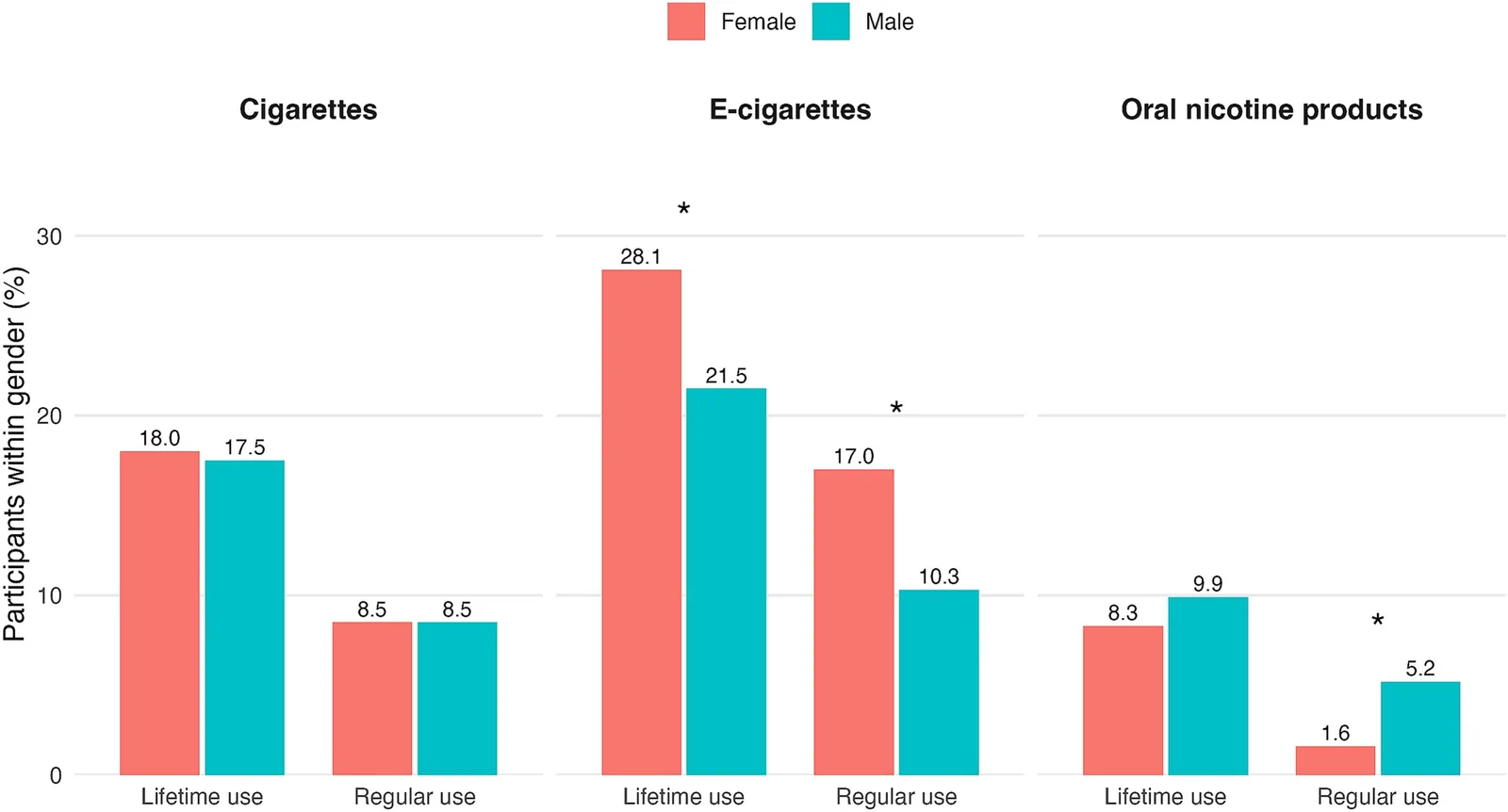
Lifetime prevalence and regular use of cigarettes, e-cigarettes, and oral nicotine products by gender among secondary school students in northern Germany, February–May 2026 (male: *n* = 446; female: *n* = 505). Bars represent the percentage of male and female participants reporting lifetime use and regular use (more than once a month) of each product. Oral nicotine products included tobacco pouches (“snus”) and nicotine pouches. * *p* < .05 (chi-square test).

In the total sample, regular cigarette use did not differ by gender (8.5% in both groups), regular e-cigarette use was more common among female than male participants (17.0% vs. 10.3%), and regular use of oral nicotine products was more common among male participants (5.2% vs. 1.6%) (Table 1). To examine whether frequency of use differed among those who had used a product, ordinal logistic regression models controlling for age were calculated among lifetime users of each product.

Ordinal logistic regression analyses controlling for age showed no significant association between gender and frequency of conventional cigarette use (OR = 0.69, 95% CI [0.38, 1.23]). Age was also not a significant predictor (OR = 0.92, 95% CI [0.76, 1.12]).

Similarly, no significant association between gender and frequency of e-cigarette use was observed (OR = 1.23, 95% CI [0.75, 2.02]). However, increasing age was significantly associated with higher frequencies of e-cigarette use (OR = 1.18, 95% CI [1.03, 1.35]). Thus, the higher prevalence of regular e-cigarette use among female participants reflected a higher proportion of girls who had ever used e-cigarettes, not a higher frequency of use among users.

For oral nicotine products, female participants reported significantly lower frequencies of use compared with male participants (OR = 0.22, 95% CI [0.08, 0.58]). Among oral nicotine product users, older age showed a trend toward higher frequencies of use, although this association did not reach statistical significance (OR = 1.39, 95% CI [0.99, 1.98]).

### 3.2 Initiation and use patterns

*E*-cigarettes were the most commonly reported first nicotine product among both male and female participants, followed by conventional cigarettes. No significant gender differences were observed regarding the first nicotine product used (Table 1).

No significant gender differences were found for the mean age at first use of conventional cigarettes or oral nicotine products. However, male participants reported a significantly lower age at initiation of e-cigarette use compared with female participants (Table 1).

Significant gender differences were observed regarding patterns of nicotine product use among adolescents with lifetime nicotine use (χ^2^(2) = 11.15, *p* = .004, Cramer's V = 0.19). Poly-use was reported more frequently by male participants than by female participants (38.2% vs. 22.2%). In contrast, female participants more often reported single-product use compared with male participants (42.5% vs. 24.5%; see Table 1).

### 3.3 Consumption contexts

For cigarette use, parties and social gatherings and use together with friends were the most commonly reported contexts for conventional cigarette use. However, solitary use was reported more frequently by male participants than by female participants (39.7% vs. 22.0%; *p* = .019). Consumption in school-related contexts was also reported more often by male participants (24.4% vs. 11.0%; *p* = .036).

Similarly, for e-cigarettes parties and social gatherings as well as use together with friends were the most frequently reported consumption contexts among both genders. No statistically significant gender differences were observed for the investigated e-cigarette consumption contexts (all *p* > .05).

For oral nicotine products, use together with friends was the most frequently reported consumption context and was more commonly reported by male participants than by female participants (70.5% vs. 35.7%; *p* = .003). Male participants also more frequently reported use in school-related contexts (29.5% vs. 7.1%; *p* = .017) and before exams or tests (32.3% vs. 6.5%; *p* = .024). Further details are presented in Fig. 2.

**Fig. 2:**
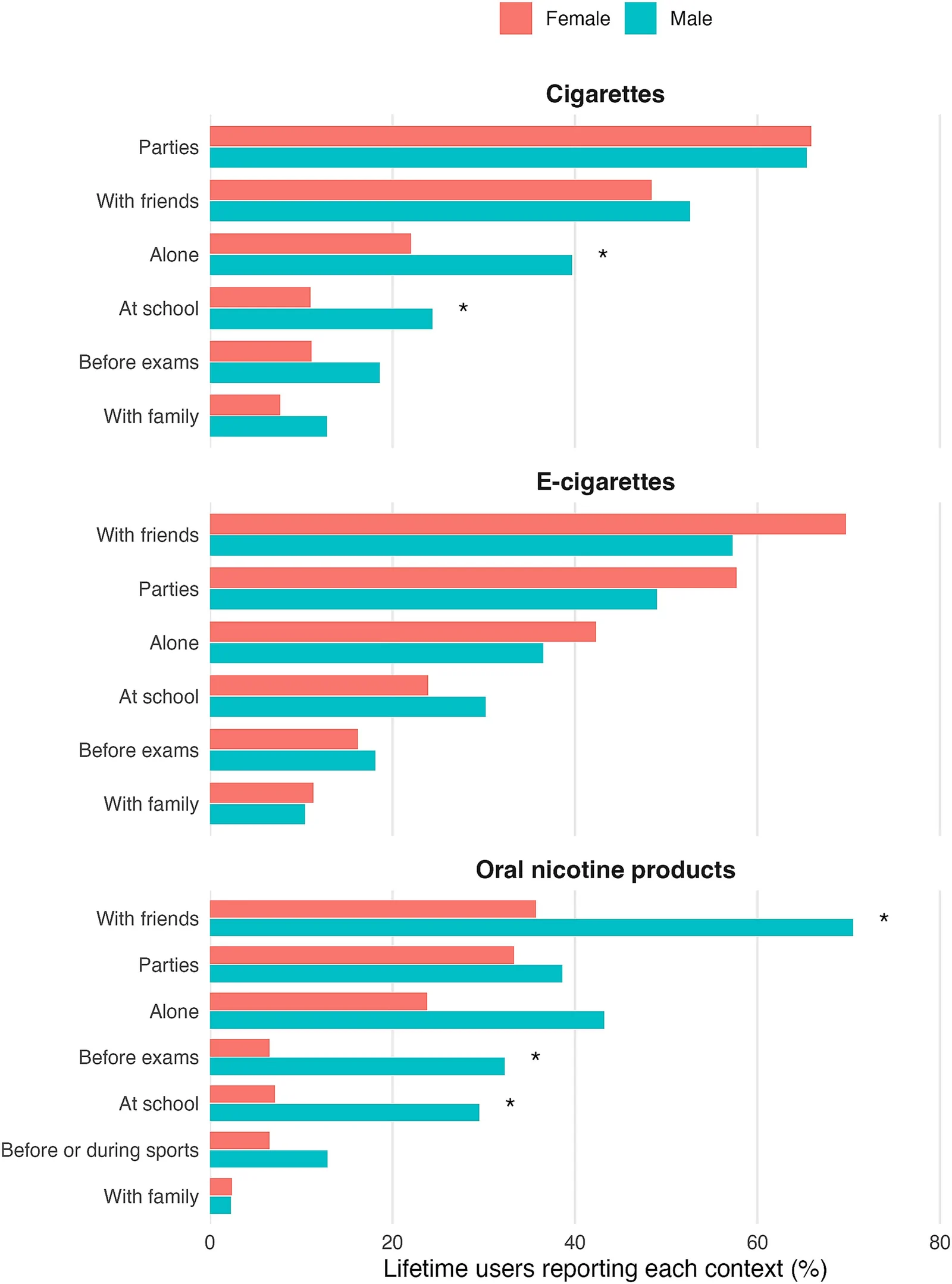
Consumption contexts of cigarettes, e-cigarettes, and oral nicotine products by gender among secondary school students with lifetime use of the respective product in northern Germany, February–May 2026. Bars represent the percentage of lifetime users of each product reporting use in each context; multiple responses were possible. The context “before or during sports” was assessed for oral nicotine products only. Sample sizes: cigarettes (male: *n* = 78; female: *n* = 91), e-cigarettes (male: *n* = 96; female: *n* = 142), oral nicotine products (male: *n* = 44; female: *n* = 42). * *p* < .05 (chi-square test).

### 3.4 Reasons for non-use and motives for use

Among adolescents reporting no nicotine use (*n* = 609), health risks were the most strongly endorsed reason against using nicotine products (male: 241 [84.9%]; female: 279 [85.8%]). Other commonly reported reasons included parental or family restrictions (male: 138 [52.9%]; female: 148 [50.7%]), perceiving nicotine products as “uncool” (male: 160 [58.2%]; female: 185 [61.1%]), and rejection of nicotine use based on personal self-image (male: 156 [60.7%]; female: 164 [54.8%]). No statistically significant gender differences were observed for any of the reported reasons against nicotine use (all *p* > .05).

Among cigarette users, stress reduction and relaxation were the most frequently reported motives for use. A significant gender difference was observed only for weight-control motives, which were reported more frequently by female than male participants (21.1% vs. 1.5%; *p* < .001).

Similarly, stress reduction and relaxation were the most commonly reported motives for e-cigarette use, whereas social and style-related motives were reported less frequently overall. No statistically significant gender differences were found for the investigated motives related to e-cigarette use (all *p* > .05).

For oral nicotine products, predominantly functional motives for use were reported. Male participants more frequently reported the perception of oral nicotine products as less harmful than conventional cigarettes as a motive for use compared with female participants (28.1% vs. 4.0%; *p* = .043). No statistically significant gender differences were observed for the remaining motives (all *p* > .05) (Fig. 3).

**Fig. 3:**
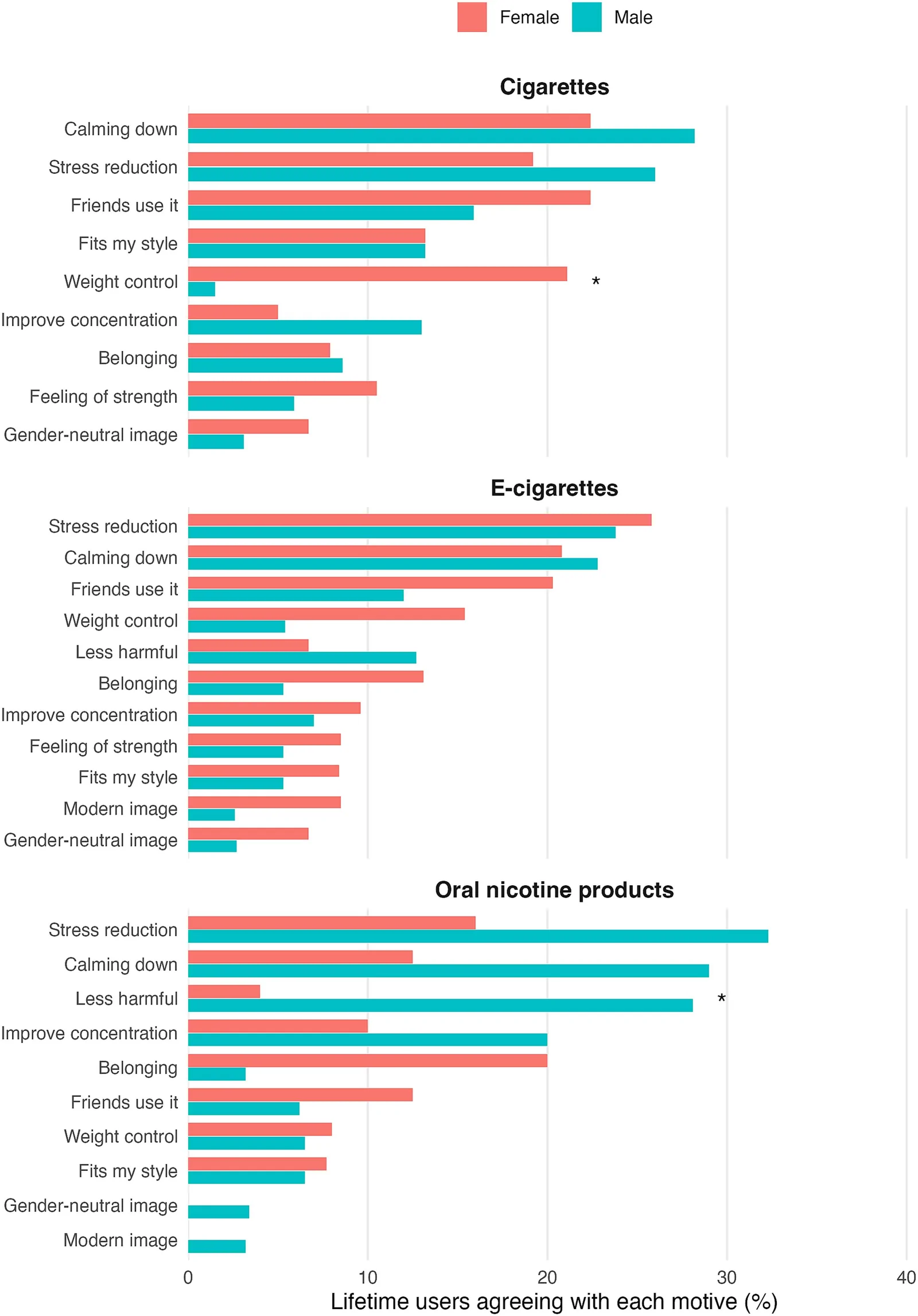
Motives for the use of cigarettes, e-cigarettes, and oral nicotine products by gender among secondary school students with lifetime use of the respective product in northern Germany, February–May 2026. Bars represent the percentage of lifetime users who agreed with each motive, defined as a rating of 4 or 5 on a five-point scale (1 = does not apply at all, 5 = fully applies). Only motives assessed for the respective product are shown, ordered from highest to lowest overall agreement. Sample sizes as in Fig. 2. *p < .05 (chi-square test).

## 4 Discussion

The present study investigated gender-specific differences in the use of modern nicotine products among adolescents and young adults. Overall, gender differences in general nicotine product use appeared less pronounced than those previously described for conventional cigarette smoking in earlier generations (Deutsches Krebsforschungszentrum and Deutsche Krebshilfe, 2025; Tanoue, 2000; U.S. National Library of Medicine, 2026). While no gender differences were observed for conventional cigarette use, female adolescents reported higher lifetime prevalence and regular use of e-cigarettes, whereas differences regarding oral nicotine products emerged primarily for regular use, patterns of use, and specific motives for consumption. These findings suggest that contemporary gender differences in adolescent nicotine use are reflected less in whether nicotine products are used overall and more in how, why, and which products are used.

This pattern was particularly evident for oral nicotine products. Although lifetime experience with oral nicotine products did not differ between genders, male adolescents reported higher frequencies of regular use and higher levels of poly-use across different products. This finding is consistent with previous research suggesting that boys may be more likely to engage in risk-oriented and experimental consumption behaviors (U.S. National Library of Medicine, 2026; Cebulla and Tomaszewski, 2009). At the same time, modern product marketing and presentation could further reinforce these patterns. Nicotine pouches in particular are often associated in social media and advertising contexts with performance, control, athleticism, and contemporary masculine identities (Ling et al., 2023). In line with this, male participants more frequently reported use in school-related contexts or for performance-related purposes, such as before exams or during sports activities. These findings may indicate that traditionally male-associated patterns of risk-taking and performance orientation continue to play a role in the context of modern nicotine products.

In contrast, female adolescents reported higher lifetime prevalence and regular use of e-cigarettes than male adolescents. Although no gender differences were observed regarding the first nicotine product used, these findings suggest that e-cigarettes may have particular appeal for female adolescents. Previous studies indicate that adolescents perceive e-cigarettes as less harmful, more modern, and more socially acceptable than conventional cigarettes (Gorukanti et al., 2017). In addition, sweet flavors, visually oriented marketing strategies, and strong visibility on social media may further increase the attractiveness of these products (McNeill et al., 2018; Reimer et al., 2016).

Another notable finding was that female participants more frequently reported weight-control motives for cigarette use compared with male adolescents. Although overall endorsement levels remained moderate, this finding may indicate that body- and weight-related motives continue to play a gender-specific role in adolescent nicotine use. This could suggest that traditional beauty ideals continue to influence health-related behaviors among adolescents despite broader social changes. Modern nicotine products may partly reproduce these mechanisms in modified forms.

Interestingly, many other motivational factors differed only marginally between genders. Social motives such as group belonging or appearing “cool” were reported relatively infrequently overall. However, these findings should be interpreted cautiously, as social dynamics related to adolescent substance use may only be partially captured through direct self-report measures. Implicit social norms, peer influences, and digital media exposure may play a greater role than standardized questionnaires are able to assess directly.

In addition, male adolescents reported an earlier initiation of e-cigarette use. Earlier initiation of tobacco and nicotine use is associated with subsequent nicotine dependence and sustained use, particularly when first use occurs at or before the age of 13 (Sharapova et al., 2020). Although the observed differences in age at initiation were relatively small, they may still be relevant from a developmental perspective.

Our findings are largely consistent with evidence from other Western countries. In US national data, exclusive e-cigarette use increased more strongly among female than male students between 2014 and 2020 (Cook et al., 2023), mirroring the higher e-cigarette use and single-product use among female participants in our study. US studies also show that oral nicotine products are frequently part of dual- and poly-use patterns among adolescents (Harlow et al., 2025; Abid et al., 2025), in line with the higher poly-use among male participants. However, comparisons are limited by differences in national regulation; for example, selected nicotine pouches are authorized for marketing in the United States (Abid et al., 2025) but cannot legally be sold in Germany. Comparisons with non-Western countries are even more limited, as regulations, product markets, and gender differences in tobacco use differ substantially; in the Western Pacific Region, for example, adult tobacco use prevalence in 2024 was 43.3% among men but 2.5% among women (World Health Organization, 2025).

Several limitations should be considered when interpreting the findings. Due to the cross-sectional design, no conclusions can be drawn regarding causality or temporal developments. Furthermore, all data were based on self-report and may therefore be affected by recall bias and social desirability bias. The sample was school-based and regionally limited, restricting the generalizability of the findings to broader adolescent populations. In addition, participants reporting diverse gender identities could not be included in inferential analyses due to small subgroup sizes, limiting the representation of gender diversity within the study.

Furthermore, tobacco pouches and nicotine pouches were assessed within a combined category of oral nicotine products, as adolescents may not clearly differentiate between these products in everyday use. Based on our observations during the school-based prevention sessions, adolescents in the study region commonly refer to nicotine pouches as “snus”; oral nicotine use in our sample may therefore mainly reflect nicotine pouch use. Nevertheless, these products differ regarding ingredients, regulation, and public perception, and future studies should examine them separately. Single, dual, and poly-use were based on lifetime use and do not distinguish experimental from current multiple product use.

Moreover, as the study was conducted in a single country with a specific regulatory framework, the findings may not be transferable to countries with different regulations, product markets, or sociocultural norms.

## 5 Conclusions

Overall, the findings suggest that gender-specific differences in adolescent nicotine use may not be disappearing, but rather changing in form. While overall prevalence rates increasingly converge between genders, gender-specific patterns remain evident regarding motives, product preferences, and consumption contexts. As adolescence represents a critical phase of identity development, young people may be particularly susceptible to gender-specific marketing strategies related to modern nicotine products. Prevention programs should therefore address modern nicotine products in a more gender-sensitive manner and increase awareness of marketing strategies and gender-related stereotypes associated with nicotine use.

## CRediT authorship contribution statement

**Mareike Lüthgen:** Writing – review & editing, Writing – original draft, Visualization, Supervision, Project administration, Methodology, Funding acquisition, Formal analysis, Conceptualization. **Florian Graf:** Investigation, Formal analysis, Data curation. **Niel Heitmann:** Project administration, Investigation, Data curation. **Adrian Heinz:** Investigation, Formal analysis, Data curation. **Daniel Drömann:** Visualization, Supervision, Project administration, Investigation, Funding acquisition. **Folke Brinkmann:** Supervision, Project administration, Investigation, Funding acquisition. **Klaas F. Franzen:** Writing – review & editing, Supervision, Project administration, Methodology, Funding acquisition, Conceptualization. **Loana Penner:** Writing – review & editing, Supervision, Project administration, Methodology, Investigation, Data curation, Conceptualization.

## Consent for publication

Not applicable. The manuscript does not contain data from any individual person in any identifiable form.

## Ethics approval and consent to participate

The study was conducted in accordance with the Declaration of Helsinki and was approved by the Ethics Committee of the University of Lübeck (reference number: AZ 2024-267). Informed consent was obtained from all participants and their legal guardians prior to participation.

## Ethics declaration

### Informed consent and patient details

Written informed consent to take part in the study and to publish the article has been obtained from all participants or their legal representatives. The privacy rights of participants have been observed.

### Studies in human

This study was performed in compliance with relevant laws, regulatory frameworks and guidelines where the research took place.

This study was approved by the Ethikkommission der Universität zu Lübeck (Approval No. 2024-267).

## Declaration of generative AI and AI-assisted technologies in the writing process

During the preparation of this work the authors used Claude (Anthropic) in order to assist with language editing and the drafting of revised text passages and the response to reviewers. After using this tool, the authors reviewed and edited the content as needed and take full responsibility for the content of the publication.

## Funding

This work was supported by the Else Jebsen und Friedrich Bluhme Stiftung, and the Niedersächsischer Verein zur Bekämpfung der Tuberkulose, Lungen- und Bronchialerkrankungen e.V. The funders had no role in the design of the study, data collection, analysis, interpretation of data, or in writing the manuscript.

## Declaration of competing interest

The authors declare that they have no known competing financial interests or personal relationships that could have appeared to influence the work reported in this paper.

## Data Availability

Data will be made available on request.
